# Case Report: Long segmental lesions of the spinal cord caused by exposure to xylene

**DOI:** 10.3389/fneur.2023.1121421

**Published:** 2023-06-15

**Authors:** Qin Du, Hongxi Chen, Ziyan Shi, Hongyu Zhou

**Affiliations:** Department of Neurology, West China Hospital, Sichuan University, Chengdu, China

**Keywords:** xylene, occupational exposure, long segmental lesions, spinal cord, case report

## Abstract

Xylene has the potential to cause nervous system disturbances since it is a lipophilic substance with high affinity for lipid-rich tissue, such as the brain. Involvement in the spinal cord, especially long segmental spinal cord lesions that permeate almost the entire cervical and thoracic spinal cord, is extremely rare. We report two cases of occupational exposure to excessive xylene, both of which presented with severe and rapidly progressive numbness and weakness in the limbs that, more importantly, led to poor outcomes: one died and the other was left severely disabled. In both, spinal magnetic resonance imaging showed long segmental lesions in the cervicothoracic spinal cord. These findings may provide some insights into the effects of xylene as an isolated agent on the spinal cord injury.

## Introduction

Xylene, a common lipophilic aromatic hydrocarbon, is widely used as a solvent in the rubber, printing and leather industries and as a thinner for paints, cleaning agents and varnishes (1). It is a colourless, sweet-smelling liquid or gas present naturally in coal, petroleum, and wood tar (2). Xylene is well-absorbed via inhalational, oral, and dermal routes, to some extent. Once absorbed, xylene enters the blood and is transported throughout the body by the circulatory system, with most of the chemical leaving within 18 h after the end of the exposure. Following prolonged exposure, particularly occupational exposure, xylene accumulates mainly in the muscles and adipose tissues (1). The toxicity of the central nervous system could be attributed to the liposolubility of xylene in the neuronal membrane, and the disturbance in the activity of the proteins that are crucial for normal neuronal function (1, 3). The neurotoxic effects observed involved impulsiveness, cancer, altered vision, behavioral changes, incoordination, seizure, elevated respiration, hyperactive to stimuli, spams, and reduce acetylcholine (4–10), while spinal cord injury related to xylene has not been reported. We present two cases of chronic occupational exposure to xylene in which both workers were in close contact with xylene and suffered from severe attacks associated with spinal cord injury after a large amount of exposure to xylene as a result of poor ventilation and improper protective measures, leading to unfavourable prognoses.

## Case presentation

### Case 1

**Figure 1:**
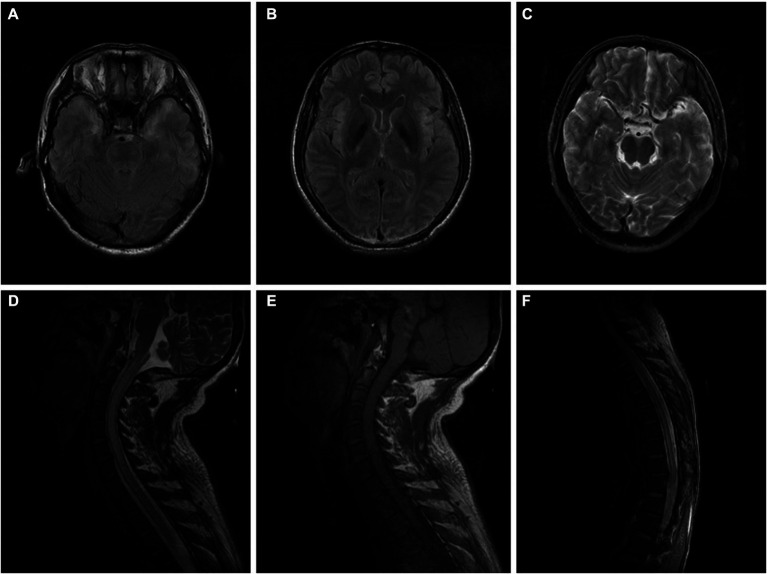
**(A–C)** Brain MRI of the patient in case 1 indicated high signal intensity of bilateral temporal lobes and insular cortex in a T2WI sequence performed without enhancement on a contrast-enhanced scan (10 days after onset). **(D–F)** Cervicothoracic MRI of the patient in case 1 showed extensive thickening of the cervical and thoracic spinal cord, especially the cervical spinal cord, and an increased signal intensity in T2WI sequence, with slightly patchy enhancement of the lesions on contrast-enhanced scan (10 days after onset).

A 48 years-old painter had worked in a small room for 4 months spraying paint in which xylene was the main component, and he often worked more than 9 h a day. Half a month before onset, the patient worked in an environment lacking ventilation and did not use adequate protective measures. He presented with mild weakness and numbness in both lower limbs. The weakness worsened rapidly, and he was unable to walk on his own within 2 days. Five days after onset, his condition progressed to paraplegia, and he was referred to the hospital. Physical examination showed hypoesthesia below the nipple. The next day, he had difficulty urinating and defecating. Seven days after onset, the patient presented with sudden loss of consciousness and generalized tonic-clonic seizure lasting for 2 min. Laboratory examinations indicated mild hypofunction of the liver. Other factors, such as serum vitamin B12 and homocysteine levels, erythrocyte sedimentation rate, myocardial markers, tumour markers, blood ammonia, Epstein-Barr virus, and TORCH (toxoplasmosis, other viruses, rubella, cytomegalovirus, herpes simplex viruses), were within normal limits. In addition, aquaporin-4 antibody (AQP4-Ab), myelin oligodendrocyte glycoprotein antibody (MOG-Ab), glial fibrillary acidic protein (GFAP) and autoimmune encephalitis antibodies were negative in his serum and cerebrospinal fluid (CSF). Immune antibodies against Sjögren’s syndrome-related antigen A (SSA) and B (SSB), antinuclear antibody (ANA) and anti-neutrophil cytoplasmic antibody (ANCA) were also negative. Brain magnetic resonance imaging (MRI) indicated high signal intensity of the bilateral temporal lobes and insular cortex on a T2WI sequence without enhancement on a contrast-enhanced scan (Figures 1A–C). Cervicothoracic MRI showed symmetrical long T1 and long T2 signals with slight enhancement on a contrast-enhanced scan (Figures 1D–F). He was treated with intravenous acyclovir (500 mg q8h) and oral levetiracetam (500 mg bid), his condition deteriorated rapidly. Eleven days after onset, he suddenly lost consciousness and fell into a coma, his blood pressure and oxygen saturation decreased progressively. Two hours later, the patient died. Unfortunately, we failed to obtain accurate pathological results of the spinal cord injury because his family refused autopsy.

His colleague who worked with him in the same environment presented with numbness of both lower limbs and instability of gait. He went to the hospital and received timely treatment, and his symptoms eventually disappeared.

### Case 2

**Figure 2:**
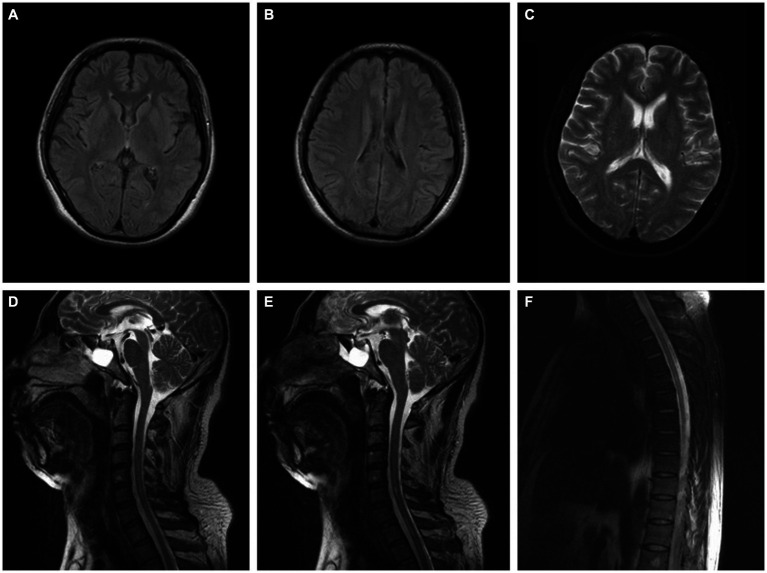
**(A–C)** Brain MRI of the patient in case 2 was normal (2 months after onset). **(D–F)** Spinal MRI of the patient in case 2 indicated longitudinal linear abnormity with long T2 signal in the cervical and thoracic spinal cord and was not enhanced on the contrast-enhanced scan (2 months after onset).

A 40 years-old man who worked as a painter for 20 years had been painting in a closed small space without protective measures for a few days. He suffered from dizziness, nausea, vomiting, and a loss of consciousness for more than an hour without epileptic seizures. He subsequently presented with involuntary movement of the right upper limb characterized by repeated gripping motions and deteriorated rapidly, later involving other limbs. This progression was accompanied by numbness and weakness. His condition was aggravated further, and within several days, he was unable to walk and take care of himself due to repeated limb spasms. Additionally, the patient frequently presented with epileptic seizures that lasted for approximately 2 to 3 min and had a slightly abnormal electroencephalogram. CSF studies were unremarkable. AQP4-Ab, MOG-Ab, GFAP and autoimmune encephalitis antibodies were negative in his serum and CSF. Electromyogram and brain MRI were normal (Figures 2A–C), whereas spinal MRI indicated longitudinal linear abnormity with a long T2 signal in the cervical and thoracic spinal cord and was not enhanced on the contrast-enhanced scan (Figures 2D–F). He was treated with intravenous methylprednisolone pulse therapy (1,000 mg/day for 5 days), intravenous acyclovir (500 mg q8h), oral clonazepam (1 mg bid) and oral levetiracetam (500 mg bid), and his symptoms partially improved. One month later, the patient presented with dysuria and incontinence accompanied by a decline in near memory and computational power, which was relieved gradually after symptomatic treatment. Eighteen months after onset, the patient still had involuntary movement and limb weakness but could walk 100 metres and eat with assistance, although paroxysmal seizures were still present. In a subsequent investigation, we found that the main component of the paint he used was xylene.

## Discussion

Xylene is a hydrocarbon solvent commonly found in a multitude of products, such as varnish, ink, paint thinners, degreasers, and insecticides (11). Xylene exposure exerts toxic effects on various systems and exposure to xylene or its isomers causes neurotoxic effects, including impulsiveness, cancer, visual and behavioural changes, memory decline, seizure, cramp, numbness, weakness and imbalance of gait (12–16). In this case study, both patients were painters and had not been previously exposed to any other neurotoxic substances. Two patients presented with manifestations of the spinal cord injury at the same time as a change in their conscious state, and both had a long-term history of exposure to xylene. Antibodies related to myelitis were not found in either patient. Furthermore, poor ventilation and a lack of effective preventive measures resulted in a larger amount of exposure. Due to the long time from onset, it was impossible to detect the final serum concentration of xylene to confirm xylene poisoning, in addition, we failed to obtain accurate pathological results of the spinal cord injury in case 1 because his family refused autopsy, therefore we cannot obtain direct evidence of spinal cord injury caused by xylene poisoning, which increased the challenge of diagnosis. It should also be distinguished from spinal cord lesions caused by other reasons. However, both showed sudden onset and rapid progression and had a history of extensive and prolonged exposure to xylene before onset, and these symptoms could not be explained by other reasons. More importantly, the colleague of the first patient also suffered from the associated symptoms. We highly suspect that the patients’ manifestations were caused by xylene poisoning, and spinal cord MRI revealed severe spinal cord injury, which is extremely rare. The damage to the nervous system caused by xylene according to previous literature was focus on brain, while little attention was paid to spinal cord injury. These two cases may provide some insights into the toxic effects of xylene on the spinal cord, but this study still has some limitations. First, in the patients in these two cases, we failed to obtain the final serum concentration of xylene to confirm xylene poisoning because of the long duration from onset, therefore, the exact causal relationship between xylene poisoning and spinal cord injury cannot be determined. Second, in the first patient, the lack of pathological results related to the spinal cord injury may have affected the accuracy of our conclusions to some degree. Further studies are warranted to improve understanding of the toxic effects of xylene and confirm our findings.

This study suggests implementing environmental monitoring systems and giving adequate focus on reducing the toxicity of xylene. Efforts to minimize the health hazards in the environment should be made to create a safer living condition by making the environmental health departments more familiar with the adverse impacts of xylene, security mechanism and contingency program. Usage of proper personal protective equipment is also vital when disposing of xylene.

## Conclusion

This is the first report of spinal cord involvement in patients following long-term exposure to xylene in the absence of any other risk factor. When patients exposed to xylene develop rapid and severe symptoms associated with the spinal cord injury accompanied by alterations in consciousness, in addition to the common causes of the spinal cord injury, the possibility of xylene poisoning should also be considered.

## Data availability statement

The original contributions presented in the study are included in the article/supplementary material, further inquiries can be directed to the corresponding author.

## Ethics statement

The study was conducted in accordance with the Declaration of Helsinki, and approved by the Ethics Committee of the West China Hospital of Sichuan University. The patients/participants provided their written informed consent to participate in this study.

## Author contributions

QD designed the study and drafted and revised the manuscript. HC and ZS interpreted the data. HZ designed the study and revised the manuscript. All authors contributed to the article and approved the submitted version.

## Funding

This work was supported by the Department of Science and Technology of Sichuan Province (2022YFS0315) and 1·3·5 project for disciplines of excellence–Clinical Research Incubation Project, West China Hospital, Sichuan University (21HXFH041).

## Conflict of interest

The authors declare that the research was conducted in the absence of any commercial or financial relationships that could be construed as a potential conflict of interest.

## Publisher’s note

All claims expressed in this article are solely those of the authors and do not necessarily represent those of their affiliated organizations, or those of the publisher, the editors and the reviewers. Any product that may be evaluated in this article, or claim that may be made by its manufacturer, is not guaranteed or endorsed by the publisher.
